# Preferences Regarding Self-Management Intervention Outcomes of Dutch Chronically Ill Patients With Limited Health Literacy

**DOI:** 10.3389/fpubh.2022.842462

**Published:** 2022-05-11

**Authors:** Marieke van der Gaag, Monique Heijmans, Marta Ballester, Carola Orrego, Ena Niño de Guzmán, Lyudmil Ninov, Jany Rademakers

**Affiliations:** ^1^Netherlands Institute for Health Services Research, Utrecht, Netherlands; ^2^Red de investigación en servicios de salud en enfermedades crónicas, Madrid, Spain; ^3^Avedis Donabedian Research Institute (FAD), Barcelona, Spain; ^4^Universitat Autònoma de Barcelona, Barcelona, Spain; ^5^Department of Clinical Epidemiology and Public Health, Sant Pau Institute for Biomedical Research, Barcelona, Spain; ^6^Ibero-American Cochrane Center (CCIb), Barcelona, Spain; ^7^European Patients Forum, Brussels, Belgium; ^8^Department of Family Medicine, Care and Public Health Research Institute, Maastricht University, Maastricht, Netherlands

**Keywords:** health literacy, chronic disease, self-management, patient preference, concept mapping

## Abstract

**Background:**

For many chronically ill patients self-management of their disease is difficult. This may be especially true for people with limited health literacy as they are faced with additional challenges in the day-to-day management of their disease. Research has shown that self-management support is most effective when tailored to the needs and preferences of patients. Therefore, this study explores the preferences regarding self-management outcomes of chronically ill patients with limited health literacy.

**Methods:**

A total of 35 patients with limited health literacy were invited to a concept-mapping procedure consisting of two card sorting tasks. Patients ranked 60 outcomes, which are often found in literature in relation to self-management, to the level that was important for themselves. Means were calculated for each outcome and domain, and differences within the group were analyzed.

**Results:**

For patients with limited health literacy, satisfaction with care is the most important outcome domain. This domain includes overall satisfaction, the communication with health care providers, the provision of information and trust. At an outcome level, outcomes related to symptom management and improving competences to self-management scored very high. No differences between patient groups for age and sex were found.

**Conclusion:**

Chronically ill patients with limited health literacy prefer a wide variety of outcomes for their self-management. Next to health related outcomes, patients mostly prefer to work on their competences for self-management. For health care professionals, acting on these patient preferences and building a solid relationship will enhance successful self-management.

## Background

The general population is getting older and the number of patients with one or more chronic disease(s) is rising (1). Chronic diseases are the leading cause of morbidity and mortality in Europe (2). In managing these chronic conditions, chronically ill patients are more often expected to play an active role in their own health care. Their role has shifted from a passive recipient of care to a more active role where patients expected to self-manage their disease (3). Also, since the last two decades in the Netherlands, a choice has been made for programmatic care for the chronically ill. There is a strong emphasis on self-management and personal responsibility of the patient and their relatives in order to relieve professional care as much as possible (4). Self-management requires people with chronic illness to undertake a variety of activities, for example, psychologically coping with their illness in daily life, changing eating behaviors, medication adherence, and communicating with their health care providers. It is therefore not surprising that many patients find it difficult to self-manage and instead experience barriers (3, 5–7). For these patients, self-management support may be needed.

Self-management support is defined as the systematic provision of education and supportive interventions to improve patients' skills and confidence in managing their health problems, including regular assessment of progress and problems, goal setting, and problem-solving support (8). During the last decades, many self-management interventions (SMIs) have been developed, aimed to equip patients (and caregivers) to actively self-manage their chronic condition(s). Previous research suggests that SMIs may be effective and lead to better patient outcomes and less health care use (9–11). Literature also shows self-management supporting interventions are more effective when they are tailored to a patient's individual needs and preferences as there is a vast variation in the extent to which patients are able and motivated to self-manage (12–14).

For the tailoring of interventions and to match the needs and opportunities of patients as much as possible, the preferences, needs and capabilities of specific patient groups and individuals should be known. All chronically ill patients would probably benefit from tailored interventions. However, a group of patients that especially may benefit are patients with limited health literacy, who constitute a large group among the general population of patients with chronic conditions (15, 16). In the Netherlands the group of chronically ill patients with limited health literacy is estimated to be 30% of the general population (17). Health literacy entails people's knowledge, motivation and competences to access, understand, appraise, and apply health information in order to make judgements and take decisions in everyday life concerning health care, disease prevention and health promotion, and in this way to maintain or improve quality of life during the life course (18). Research has shown that limited health literacy levels are associated with poorer self-management skills (19), poorer health outcomes, and increased health care use (20, 21). Patients with limited health literacy often face additional difficulties during the management of their illness, as they may lack the right knowledge and information to make decisions about their health, or the skills to get or apply that information. Also, more than other patients, patients with limited health literacy often lack self-confidence to ask questions to a caregiver, and sometimes lack motivation to live healthier (22–24).

Qualitative research, exploring patients needs and preferences shows that a variety of outcomes are important for chronically ill patients in the context of or as a result of self-management (3, 25–28). Often they aim for better medical management of the disease like a reduction in daily symptoms or better adherence to medical advice. However, patients also wish to make lifestyle changes, get more satisfaction from their treatment, have better interaction with their health care providers, and a better quality of life and well-being. In addition, intermediate SMI outcomes are also mentioned as important goals to strive for. These intermediate outcomes can be considered to be prerequisites for successful self-management (support) and may be related to the patient's knowledge, self-efficacy, motivation, behavioral skills or the process of care, such as a trustful relationship with health care providers or more continuity in care offered by different health care proffesionals.

It is important to know which outcomes are especially important for patients with limited health literacy as this insight may help to better support this vulnerable group in their self-management and may help for the future development and evaluation of self-management interventions directed to this large patient group. Evidence of effectiveness should derive from trials that assess outcomes that are important to patients. Besides, identifying important outcomes from the patient perspective, and taking these as a starting point for intervention development, contributes to uniformity and standardization of outcome reporting (29).

Since preferred outcomes with respect to self-management of patients with limited health literacy have not yet been extensively studied, the aim of this study is to explore which outcomes of self-management are most important for chronically ill patients with limited health literacy and whether these outcomes differ according to patient characteristics such as sex, comorbidity or age.

## Methods

To explore which outcomes are most relevant for patients with limited health literacy in the context of or as a result of self-management, we build on experiences and results from an ongoing international study about effective Self Management Interventions (SMIs) for patients with chronic illnesses. In this study, called COMPAR-EU (30), a general catalog of SMI outcomes used in self-management interventions for patients with chronic diseases was developed and structured, based on an extensive literature review and expert opinions. In this catalog, SMI outcomes are categorized in seven domains (see Box 1) (27, 28). Each domain contains both outcomes that are generic across chronic diseases (e.g., symptom monitoring within the domain of competences and self-monitoring) and disease specific outcomes that can be added when used for a specific diseases (e.g., monitoring blood glucose in case of diabetes). Within COMPAR-EU these seven domains were specified for patients with type 2 diabetes, COPD, heart failure, and obesity. All four diseases together resulted in a catalog including 145 different SMI outcomes, including both generic and disease-specific outcomes. This catalog of outcomes was used as the starting point of this study. To explore which outcomes are most relevant for SMIs from the perspective of patients with limited health literacy, we used a concept-mapping approach consisting of two card sorting tasks. The preparation and execution of these tasks is described below.

Box 1

**Seven domains of SMI outcomes**
- Competences and self-management behaviours
- Health related aspects
- Quality of life of patients
- Caregivers' quality of life and competences
- Satisfaction with care
- Health care use
- Costs

### Recruitment

Patients with limited health literacy were recruited using an advertisement distributed by local organizations such as an organization for people with reading and writing difficulties, online peer support groups for patients with diabetes and COPD, and patient organizations in the Netherlands. These organizations contacted there members via their newsletters or social media channels. The advertisement was written in simple Dutch language and invited people to participate in our study who encountered difficulties in their interaction with health care or during the daily management of their disease. Examples were given such as: problems to understand their doctor or medical information, difficulties in following medical advise, or difficulties in finding the right health care. In the advertisement some examples of possible problems were given inspired by the Single Item Literacy Screener (31) and the brief questions of Chew, which are both frequently used and validated to screen for limited health literacy (32). Patients who felt addressed by this advertisement could sign up as a participant of this study by contacting the researchers by email or phone in February and March 2020. In addition to the experience of problems in using or finding health care, people had to be 18 years or older and reported one or more self-reported diagnoses of a chronic disease.

### Outcomes Preparation

To explore which outcomes are most relevant for SMIs for patients with limited health literacy, we used the catalog of outcomes developed in the COMPAR-EU study for four specific diseases as the starting point. The 145 different outcomes of this catalog consisted of both generic and disease-specific outcomes. Since this current study explores self-management outcome preferences relevant for patients with limited health literacy suffering from different chronic conditions, we merged and rephrased the disease-specific outcomes in generic terms. This procedure was done independently by two researchers. For example, dietary habits comprising “minimizing water consumption” for heart failure and “adherence to dietary habits” for diabetes and COPD were merged and rephrased in one single outcome “dietary habits.”

Furthermore, specific terms which slightly differed were merged. For example terms like “Weight loss,” “Bodyweight,” and “Stable weight” were merged to “Weight control.” Outcomes as “short-term COPD symptoms” and “short term heart failure symptoms” were merged and called “short term symptoms.” This process resulted in a list of 60 generic outcomes relevant in the context of chronic disease SMIs. All outcomes were translated to Dutch and formulated in plain language by MG and checked by MH. MH was also involved in the development of the catalog of outcomes in COMPAR-EU so they knew the meaning of each outcome very well. MH is an expert in health literacy and has vast experience in writing texts for this target group or translating difficult words into plain language.

### Concept Mapping

At the start of this study, which took place during the first wave of COVID-19, we planned to hold two face to face group meetings in which we wanted to explore and rank outcomes of SMIs to the extent that they are important for people with limited health literacy. Concept mapping is a frequently used method to discuss complex topics in a structured way. In general concept mapping consists of two rounds of card sorting tasks. During the first task, participants group outcomes in concepts and subsequently rate the outcomes by importance. This method has been often used to explore patient preferences and is a highly valued method to discuss complex topics in a structured way (33–36).

The intention for this study was to use a concept mapping approach consisting of the two card sorting tasks. At first, the participants received instructions to group the cards by concepts. Unfortunately, during the face-to-face sessions, it appeared that the participants were unable to perform this task. It turned out to be too complex for them to sort 60 outcomes according to the similarity of contents. Participants were unable to look at outcomes on a conceptual level and only looked at whether the outcome was relevant for them personally. Therefore, after the face-to-face sessions (*n* = 6), we decided to only use the prioritization card sorting task, which was easier to perform. During this card sorting task, the participants individually sorted the outcome cards based on the importance they personally attached to an outcome. During the face-to-face sessions it became clear that the participants understood the wording of the outcomes correctly. The main question during the card sorting task was: “*What's most important for you? For the self-management of my disease, I would like to…”* Outcomes were phrased like “to take my meds properly,” or “to be satisfied with my care” or “not feel anxious.” For this task, the following rules applied: all outcomes had to be placed in one of five piles, from 5 (most important) to 1 (not important at all); and outcomes had to be distributed equally across the five piles, thus requiring patients to think and set priorities about differences in importance. The card sorting task was carried out in two different ways: initially in face-to-face meetings with participants using an actual pile of cards. The face-to-face meetings took place at a location of choice of the participants and were performed by MG and MH. Later, because of the restrictions by COVID-19, the data was collected digitally using the software of Provenbyusers, which is user-friendly program (https://provenbyusers.com). In the latter, the participants got a link to the online software, where they virtually could divide the pile of cards in the appropriate categories. The participants had the option to contact MG when having difficulties with accessing the online software. For the first five online participants there was a quick telephone follow-up to make sure the card sorting task was understood correctly and to address digital difficulties. All participants filled in a short questionnaire upfront, to collect their demographics, such as: sex, age and chronic disease. The patients who participated face-to-face signed a written informed consent, and the online participants gave their consent via email. Participation was anonymous and participants had the option to withdraw from the study at anytime.

For this study no ethical clearance from a recognized medical ethics review committee was necessary. According to the Medical Research Involving Human Subjects Act (WMO), this study does not influence the research participants' health care they receive.

### Analyses

Means and standard deviations were calculated for each outcome separately, and for all domains, using STATA version 15.0. Results were grouped and presented per domain and by a top-15 of individual outcomes. Missing values were replaced by the mean value of the outcome. Participants who performed the card sorting task incorrectly, for example by answering almost all outcomes “important” and who did not try to equally divide the outcomes over all answering options were excluded. *T*-tests were used for the analysis of differences among groups defined by sex and comorbidities. Age was analyzed as a continuous variable, using Pearson's correlation coefficient. Differences were considered significant with a *p*-value <0.05.

## Results

### Participant Characteristics

In total, 39 patients participated in the card sorting task, of which 35 were included in the analysis. Four participants were excluded due to incorrectly performing the card sorting task. Every participant was diagnosed with one or more chronic diseases. Twenty patients suffered from more than one chronic diseases (*n* = 20). The diseases reported most were COPD, asthma, diabetes and cardiovascular diseases. Mean participant age was 66 years and 54% of the participants were female. All face-to-face participants were female, but there were no significant differences in mean age or distribution of comorbidities between the online and face-to-face group. A summary of the participant characteristics is described in Table 1.

**Table 1:** Participant characteristics.

		**Participants (*n =* 35)**
Age, mean (sd), range		65.9 (10.1), [36–89]
Gender	Male, *n* (%)	16 (46)
	Female, *n* (%)	19 (54)
Mode of participation	Online, *n* (%)	29 (83)
	Face to face, *n* (%)	6 (17)
Comorbidities	No, *n* (%)	15 (43)
	Yes, *n* (%)	20 (57)

### Preferences at Domain Level

An overview of all scores of the domains and items is presented in Table 2. Patients with limited health literacy rated the domain “satisfaction with care” as most important with a mean (SD) of 3.44 (0.77). This domain describes care satisfaction overall and the relationship between the health care professional and the patient, including trust, communication and getting enough information. Subsequently, “health related aspects,” mean 3.25 (0.38), are also important for patients with limited health literacy. In this domain, mainly outcomes related to “seriousness of the disease” and “disease management,” score high, but mortality on the contrary, rated low with a mean of 1.89 (1.11). “Symptom control” has the highest mean score with a mean of 4.23 (0.91), followed by “being in good shape.” Outcomes in the domain “patients' competence in self-management behaviors” score a mean of 3.10 (0.30). The highest scoring outcome in this domain is medication adherence (mean 4.06), followed by patient activation (mean 3.80) and self-efficacy (mean 3.80). Self-management competences also include two outcomes on health literacy. “How to find health information” scores 2.8 (1.02) and “How to use health information” scores 2.63 (1.17). The mean scores of the domains did not differ significantly by mode of participation.

**Table 2:** Mean importance scores of the seven outcome domains and their outcomes.

	**Mean (sd)**
**Satisfaction with care**	**3.44 (0.77)**
Health care professional trust	3.57 (1.20)
Communication with health care professionals	3.54 (1.20)
To get enough information from health care professional	3.46 (1.22)
Care satisfaction	3.20 (1.16)
**Health related aspects**	**3.25 (0.38)**
*Seriousness of the disease*	*3.51 (0.53)*
Being in good shape	4.14 (0.91)
Exercise capacity	3.77 (1.26)
Reduce the chance of developing other disease	3.34 (1.41)
General metabolic functions	2.80 (1.32)
*Disease-management*	*3.49 (0.50)*
Symptom control	4.23 (0.91)
Symptom recognition	3.74 (1.04)
Sleep quality	3.69 (1.32)
Fatigue	3.69 (1.18)
Prevent progression of symptoms	3.66 (1.43)
Maintaining healthy nutrition	3.57 (1.20)
Maintaining physical activity	3.43 (1.38)
Blood pressure control	3.06 (1.37)
Weight management	2.94 (1.39)
Pain	2.91 (1.54)
*Complications*	*2.21 (0.99)*
Cholesterol	2.40 (1.19)
Hyperglyceamia	2.03 (1.25)
*Mortality*	*1.89 (1.11)*
Lower risk of death	1.89 (1.11)
**Patients competence in self-management behaviors**	**3.10 (0.30)**
*Self-management competences*	*3.30 (0.47)*
Patient activation	3.80 (1.28)
Self-efficacy	3.80 (1.18)
Participation and decision making	3.74 (1.31)
Knowing what care I am entitled to	3.09 (1.36)
Knowledge	3.00 (1.14)
To take my own decisions together with my family	2.86 (1.38)
Health literacy/ how to find health information	2.80 (1.02)
Health literacy/ how to use health information	2.63 (1.17)
*Self-management/self-care behaviors*	*2.96 (0.41)*
Medication adherence	4.06 (1.03)
Physical activity	3.74 (1.24)
Dietary habits	3.69 (1.25)
Adherence to program	3.17 (1.25)
Self-monitoring	3.14 (1.29)
Alcohol consumption	1.6 (0.85)
Smoking cessation	1.31 (0.76)
**Quality of life**	2.92 (0.33)
*Psychological functioning*	*3.21 (0.63)*
Positive attitude	3.97 (1.12)
Happiness	3.60 (1.31)
Coping	3.54 (1.22)
Stress	3.28 (1.41)
Participation in social activities	3.17 (1.52)
Depression	2.77 (1.52)
Hostility	2.71 (1.13)
Anxiety	2.60 (1.33)
*Treatment burden*	*2.82 (2.92)*
Organize my own care	3.17 (1.40)
Limit treatment side effects	2.94 (1.14)
Medication burden as a perception	2.94 (1.28)
Treatment burden in terms of time	2.23 (1.09)
*Social relations and acitivities*	*2.66 (0.33)*
Family relationships	3.11 (1.35)
Meeting other patients	2.20 (1.16)
*Physical functioning*	*2.64 (0.45)*
Being able to do the things I want to	3.86 (1.22)
Mobility	3.60 (1.38)
Being able to do sports	2.37 (1.29)
Being able to work	1.74 (1.09)
Sex life	1.63 (0.97)
**Health care use**	**2.41 (0.88)**
Number of visits/contacts with health care provider	2.51 (1.12)
Number of hospital admissions	2.31 (1.37)
**Caregiver quality of life and competences**	**2.34 (0.87)**
Caregiver knowledge	2.46 (1.09)
Caregiver burden	2.23 (1.42)
**Health care costs**	**2.17 (0.98)**

*The Bold values are the headings of the 7 outcome domains and their mean value*.

### Preferences at Outcome Level

Table 3 presents the top-15 of highest scoring outcomes. Three outcomes have a mean importance >4, which corresponds to “very important.” “Symptom control” has the highest mean score, followed by “being in good shape,” and “medication adherence.” The table shows that outcomes that were important to chronically ill patients with limited health literacy are diverse and belong to a variety of outcome domains.

**Table 3:** Top-15 outcome mean scores.

**Outcome**	**Mean (sd)**	**Domain**
Symptom control	4.23 (0.91)	Health related
Being in good shape	4.14 (0.81)	Health related
Medication adherence	4.06 (1.03)	Patients' competences
Positive attitude	3.97 (1.12)	Quality of life
Being able to do the things I want to	3.86 (1.22)	Quality of life
Self-efficacy	3.80 (1.18)	Patients' competences
Patient activation	3.80 (1.28)	Patients' competences
Exercise capacity	3.77 (1.26)	Health related
Symptom recognition	3.74 (1.04)	Health related
Participation and decision making	3.74 (1.31)	Patients' competences
Physical activity	3.74 (1.24)	Self-care behaviors
Fatigue	3.69 (1.18)	Health related
Sleep quality	3.69 (1.32)	Health related
Dietary habits	3.69 (1.25)	Self-care behaviors
Prevent progression of symtoms	3.66 (1.43)	Health related

### Difference in Outcome Preferences by Background Characteristics

The mean scores of each outcome domain by sex, comorbidity, and age are presented in Table 4. Although no significant differences were found concerning sex and age, there were certain trends in the mean scores. The highest scoring domains for males are subsequently “Health related aspects” (mean 3.33), “Satisfaction with care” (mean 3.28) and “Competences and self-management behaviors” (mean 3.07). The ranking of highest scoring domains slightly differed for females, namely “Satisfaction with care” (mean 3.58), “Health related aspects” (mean 3.19) and “Competences and self-management behaviors” (mean 3.12). A Pearson's correlation was run to assess the relationship between age and the mean scores of outcome preferences. No correlation was found between age and the outcome domains. For participants with comorbidities, the mean score of “Caregivers quality of life and competences” was significantly lower compared to participants with no comorbidities.

**Table 4:** Mean scores of outcome preferences by background characteristics.

	**Total group**	**Sex Mean (SD)**		**Comorbidity Mean (SD)**			**Age Pearson's correlation**		
		**Men (n = 16)**	**Women (n = 19)**	**P-value**	**No (n = 15)**	**Yes (n = 20)**	**P-value**	**r (33)**	**P-value**
Competences and self-management behaviors	3.10 (0.30)	3.07 (0.29)	3.12 (0.31)	0.60	3.20 (0.32)	3.02 (0.25)	0.07	−0.03	0.85
Health related aspects	3.25 (0.38)	3.33 (0.36)	3.19 (0.39)	0.29	3.16 (0.34)	3.32 (0.40)	0.24	0.09	0.61
Quality of life of patients	2.92 (0.33)	2.84 (0.30)	2.99 (0.34)	0.19	2.81 (0.27)	3.00 (0.35)	0.09	−0.11	0.52
Caregivers quality of life and competences	2.34 (0.87)	2.28 (0.93)	2.39 (0.84)	0.71	2.70 (0.88)^*^	2.08 (0.78)^*^	0.03	0.03	0.87
Satisfaction with care	3.44 (0.77)	3.28 (0.75)	3.58 (0.79)	0.26	3.47 (0.83)	3.43 (0.74)	0.88	0.06	0.75
Health care use	2.41 (0.88)	2.50 (0.89)	2.34 (0.88)	0.60	2.53 (0.95)	2.33 (0.83)	0.50	0.06	0.73
Costs	2.17 (0.98)	2.19 (0.83)	2.16 (1.12)	0.93	2.00 (0.85)	2.30 (1.08)	0.38	−0.01	0.93

* *P-value < 0.05*.

## Discussion

Until now, information about outcome preferences of patients with limited health literacy regarding SMIs was limited. This study shows that, patients with limited health literacy rated a large number of outcomes as important. These outcomes belonged to a variety of outcome domains. Consistent with the literature on chronically ill patients in general, medical outcomes like indicators of symptom control, being in good shape and medication adherence are among the outcomes that are perceived as most important by patients with limited health literacy. These outcomes are logical consequences of treatment guidelines and often the first goals of professional care in chronic conditions, such as: diabetes, cardiovascular diseases, and lung diseases. This study shows that also for patients with limited health literacy these outcomes are important. However, when asking patients what they think are important outcomes, literature shows that outcomes are often much broader. Although patients also strive for good medical outcomes and a healthy lifestyle, they also strive for a good quality of life for themselves and their network, participation in work, shared decision making, autonomy, and an active role in their own care (26, 37). This was shown in a recent European study, COMPAR-EU (30), in which the outcome preferences of chronically ill patients with diabetes, heart failure, obesity, and COPD were studied. The catalog of outcomes for these studies were the same as we used as a starting point for this study (27). During a Delphi exercise (unpublished data), patients with diabetes, COPD, heart failure, or obesity also ranked the outcomes according to importance. Although the methods are not completely comparable (items were in English, not simplified, and scoring was per item on a 10-point scale), it is interesting to compare their results with the results of this study as both study results are from a patient's perspective. Our highest scoring outcomes, especially adherence and symptom control, correspond with the results of the Delphi study in COMPAR-EU. However, the domain of satisfaction with care scored solely highest in the group of patients with obesity. Patients with heart failure and COPD ranked the domain of competences in self-management the highest, and for patients with diabetes the highest domains were the health-related outcomes.

This is not different for people with limited health literacy as we noticed in this study. Besides the frequently used health related outcomes, also quality of life related outcomes, and outcomes related to the patient-professional relationship were rated as important. Overall, at domain level, the results of this study show that patients with limited health literacy rank the domain of satisfaction with their care as most important. Satisfaction with care includes having trust in on own's health care professional and good communication and information provision. Besides that, outcomes related to the patients competences were seen as essential. Having trust in one's own competences (self-efficacy) and being able to play an active role in their own treatment (patient activation) were on the highest scoring items.

It is not surprising that patients with limited health literacy highly rank communication support for their self-management, as these patients often lack these skills. Communication skills are crucial for patients to have an active role and for shared-decision making, and to successfully deal with the daily management of a chronic disease. The same goes for self-management competences like self-efficacy and patient activation, which are prerequisites for successful self-management. However, this is often difficult for patients with limited health literacy as they lack the necessary skills for this due to limited knowledge, reading problems, or difficulties in understanding complex information. Self-management often concerns applying advices from health care professionals, independently at home. This requires knowledge and behavioral skills. As patients with limited health literacy often lack these skills, an intermediate step is required. Advices need to be understood first, as well as skills learned. This intermediate step is often automatically successful in patients having adequate health literacy, but not in patients with limited health literacy. A recent scoping review studying patient preferences of self-management, also based on qualitative studies, confirms that patients especially value the relationship with their health care professional. Empathy, emotional support, and compassionate care enhance the adherence to self-management tasks (37).

We did not find significant differences within our group of patients with limited health literacy according to sex, age, or comorbidity. This may be due to the small number of people participating and is in contrast with the findings in other studies. The results seemed to show certain trends, however these are consistent with quantitative studies: for example for participants with comorbidities the mean score of “caregivers quality of life and competences” was significantly lower, but this needs to be further explored with larger sample sizes. It is known that self-management aspects, like motivation and confidence, often differ between patient groups with different background characteristics. For example, literature has shown that men and women differ in self-confidence and motivation to self-manage, and have different preferences regarding patient-provider communication (38, 39) as women often prefer a more active role during consultation than men; the same counts for younger people compared to older (40–42). Having comorbidities also makes optimal self-management harder to achieve. Patients with comorbidities often get various advices from different health care providers, which also can be contradictory (43–45).

Our work is subject to some limitations. The Dutch study sample may not be entirely representative for the population of patients with limited health literacy in the Netherlands. We know that about 10% of the people with limited health literacy in the Netherlands have problems with reading and some have problems with using a computer. Due to the online card sorting method, patients who lack these skills might have not signed up for this study. However, all participants applied to this study because they experienced difficulties in obtaining or understanding information or in their access to health care to some extent. The online software could also have resulted in invalid results of the card sorting task due to a lack of digital literacy skills. The software, however, was so intuitive that digital errors were unlikely. This was ensured by the telephone follow-up with the first participants. In addition, we had a rather small group of patients as this was an exploratory study and recruiting patients was challenging during the COVID-19 pandemic. It is plausible that the expected differences between patient groups were not found due to limited sample sizes or the heterogeneity of chronic illnesses of the sample. For future research, we suggest larger sample sizes and also to include patients that have difficulties in reading. This would also mean another approach, for example with face-to-face interviews, with the advantage of obtaining qualitative information on why certain outcomes are important for patients.

### Implications for Policy and Practice

The most important implication for clinical practice is that since patients with limited health literacy prefer a variety of outcomes for their self-management, it is important for health care professionals to explore these preferences together with the patient in advance. In addition, patients with limited health literacy need specific attention for the prerequisites of self-management, for example knowledge, self-efficacy, and learning self-management competences, before focusing on health related outcomes. A solid relationship between the patient and the health care professional will enhance this process to successful self-management.

During the initially planned concept mapping approach, it became clear that participants were unable to think in an abstract way about the self-management concept, beyond their own experiences. The inability of patients to understand and apply abstract goals is an important implication for clinical practice. Health care professionals should tailor their communication about care and self-management to the specific individual situation of the patient to be fully understood and pay time and effort to explain how outcomes relate to each other.

## Conclusion

In summary, chronically ill patients with limited health literacy prefer a wide variety of outcomes for their self-management and differ in this way not from the average patient with a chronic disease. However, patients with limited health literacy prefer more than others to work on their competences for self-management. For health care professionals, acting on these patient preferences and building a solid relationship will enhance successful self-management.

## Data Availability Statement

The raw data supporting the conclusions of this article will be made available by the authors, without undue reservation.

## Ethics Statement

Ethical review and approval was not required for the study on human participants in accordance with the local legislation and institutional requirements. The patients/participants provided their written informed consent to participate in this study.

## Author Contributions

MG contributed to the design of the project and prepared the manuscript of the article. MH, MB, CO, EN, LN, and JR contributed to the design of the project and reviewed and contributed to the manuscript of the article. MH, MB, and CO contributed to the catalog of outcomes used as a starting point for this study. All authors contributed to the article and approved the submitted version.

## Funding

This work was supported by European Union's Horizon 2020 research and innovation programme under grant agreement No 754936.

## Conflict of Interest

The authors declare that the research was conducted in the absence of any commercial or financial relationships that could be construed as a potential conflict of interest.

## Publisher's Note

All claims expressed in this article are solely those of the authors and do not necessarily represent those of their affiliated organizations, or those of the publisher, the editors and the reviewers. Any product that may be evaluated in this article, or claim that may be made by its manufacturer, is not guaranteed or endorsed by the publisher.
